# 2-Cys Peroxiredoxins: Emerging Hubs Determining Redox Dependency of Mammalian Signaling Networks

**DOI:** 10.1155/2014/715867

**Published:** 2014-02-04

**Authors:** Jinah Park, Sunmi Lee, Sanghyuk Lee, Sang Won Kang

**Affiliations:** ^1^Korean Bioinformation Center, KRIBB, Daejeon 305-806, Republic of Korea; ^2^Department of Life Science and Research Center for Cell Homeostasis, Ewha Womans University, 52 Ewhayeodaegil, Seodaemun-gu, Seoul 120-750, Republic of Korea; ^3^Global Top 5 Research Program, Ewha Womans University, 52 Ewhayeodaegil, Seodaemun-gu, Seoul 120-750, Republic of Korea

## Abstract

Mammalian cells have a well-defined set of antioxidant enzymes, which includes superoxide dismutases, catalase, glutathione peroxidases, and peroxiredoxins. Peroxiredoxins are the most recently identified family of antioxidant enzymes that catalyze the reduction reaction of peroxides, such as H_2_O_2_. In particular, typical 2-Cys peroxiredoxins are the featured peroxidase enzymes that receive the electrons from NADPH by coupling with thioredoxin and thioredoxin reductase. These enzymes distribute throughout the cellular compartments and, therefore, are thought to be broad-range antioxidant defenders. However, recent evidence demonstrates that typical 2-Cys peroxiredoxins play key signal regulatory roles in the various signaling networks by interacting with or residing near a specific redox-sensitive molecule. These discoveries help reveal the redox signaling landscape in mammalian cells and may further provide a new paradigm of therapeutic approaches based on redox signaling.

## 1. Introduction

It is generally accepted that the cellular antioxidant enzymes belong to a group of the oxidoreductase enzymes maintaining the cellular redox homeostasis. However, the importance of antioxidant enzymes is given a spotlight after a paradigm shift of the cellular function of reactive oxygen species (ROS) from toxic respiratory by-products to a signaling second messenger. Peroxiredoxin (Prx) is a family of antioxidant enzymes exhibiting peroxidase activity which reduces the hydroperoxides to water in the presence of proper electron donors. Prxs are classified by the number of cysteine residues involved in the peroxidase activity: 2-Cys Prxs and 1-Cys Prx. The 2-Cys Prxs form a disulfide bond by reacting with the peroxides and the disulfide is reduced by thioredoxin which is coupled with thioredoxin reductase and NADPH. Therefore, 2-Cys Prxs are the first thioredoxin-dependent peroxidase enzymes [1, 2]. The 2-Cys Prxs are purely cysteine-based peroxidase enzymes with no cofactor or selenocysteine requirement. They are divided into typical and atypical groups based on the catalytic mechanism. Typical 2-Cys Prxs (Prx1–Prx4) are active as dimers organized in antiparallel fashion: that is, the peroxidatic cysteine residue (*C*_*P*_) in the amino terminus of one subunit reacts with the hydroperoxides and the resulting *C*_*P*_ sulfenic acid forms a disulfide linkage with the sulfhydryl group of resolving cysteine residue (*C*_*R*_) in carboxyl terminus of another subunit [3]. In contrast, an atypical 2-Cys Prx (i.e., Prx5) catalyzes the H_2_O_2_ reduction reaction through the formation of intramolecular disulfide linkage [4]. The 2-Cys Prx enzymes have distinct roles in diverse cellular processes, such as proliferation, migration, apoptosis, and metabolism, and are fundamentally supported by a broad distribution of the isoforms throughout the subcellular compartments. For example, Prx1 and Prx2 are the most abundant antioxidant enzymes in cytosol. Prx3 is a major mitochondrial peroxidase responsible for efficient elimination of H_2_O_2_, which is continuously produced by the dismutation of superoxide anions formed as a result of a partial reduction of the dissolved oxygen molecules during mitochondrial respiration. Prx4 is in both endoplasmic reticulum (ER) and extracellular fluid. Recent studies indicate that Prx4 is involved in the oxidative protein-folding pathway by the reoxidation of protein disulfide isomerase [5, 6]. The distribution of Prx5 is somewhat complex: high in mitochondria, some in peroxisome, and low in cytosol [4]. Hence, the cellular abundance and broad distribution of 2-Cys Prxs mark them as a major antioxidant system in mammalian cells.

Beside their primary function as antioxidant enzymes, the observation that 2-Cys Prxs peroxidase activity can be readily inhibited by overoxidation of the active site cysteine residue (*C*_*P*_) and reactivated by sulfiredoxin-dependent reduction [7] highlights novel and unforeseeable functions of these enzymes. In both *in vitro* enzyme reaction with high concentration of H_2_O_2_ and oxidatively-stressed cells, the *C*_*P*_-sulfenic acid at the active site of typical 2-Cys Prxs is overoxidized to sulfinic/sulfonic acids [8]. Unlike bacterial homologs, the typical 2-Cys Prxs in eukaryotes have been characterized to show a structural feature that the resolving cysteine (*C*_*R*_) buries away in latent enzyme and then reacts with *C*_*P*_-sulfenic acid by local unfolding of the C-terminus [9]. It is therefore interpreted that such conformational change of the C-terminus in eukaryotic 2-Cys Prxs necessary for forming a disulfide linkage with the *C*_*P*_-sulfenic acid tolerates an additional reaction of the *C*_*P*_-sulfenic acid with the second molecule of H_2_O_2_. Consequently, the 2-Cys Prxs can be inactivated by overoxidation during the reaction cycle and, if the inactive enzymes are accumulated, the local H_2_O_2_ concentration may be raised (“Floodgate” hypothesis).

Subsequent studies state that the overoxidation of *C*_*P*_ most probably corresponds to a gain of function of 2-Cys Prxs in eukaryotes. The first surprising result is that the overoxidized 2-Cys Prxs are multimerized and function as a molecular chaperone to prevent unfolded proteins from irreversible aggregation [10]. Hence, the evolution of the eukaryotic 2-Cys Prxs sensitive to overoxidation implies a highly efficient survival tactic in eukaryotes adapting oxidative stress. Recently, Veal and her colleagues reported that the overoxidation of 2-Cys Prxs plays a role in cell survival other than as a molecular chaperone [11]. In this study, the inactivation of 2-Cys Prx by overoxidation discharged a key coupling redox protein, thioredoxin, which in turn rescued other oxidized client proteins by reduction. Another compelling biological role of the 2-Cys Prx overoxidation is a correlation with circadian rhythm in normal physiology. O'Neill and Reddy have shown that the overoxidation of 2-Cys Prxs exhibits a circadian oscillation with a period of about 24 hours in human red blood cells [12]. Later, it turned out to be a transcription-independent circadian marker universally conserved from bacteria to eukaryotes [13]. Consequently, the intrinsic susceptibility of 2-Cys Prxs to inactivation by overoxidation is seemingly to be a part of the important redox mechanism in both normal and abnormal physiology. In addition, a study using yeast mutant strains lacking multiple thiol peroxidases including all five Prxs and three glutathione peroxidase genes suggests that the thiol peroxidases may transfer the ROS signals to gene expression by transcriptional regulation [14]. Therefore, in this review, we collect the evidence for specific signaling functions of typical 2-Cys Prxs with low *K*_*m*_ for H_2_O_2_ and discuss its implication as a conceptually new hub in signaling networks.

## 2. 2-Cys Prxs in Protein Phosphorylation Signaling Networks

**Figure 1:**
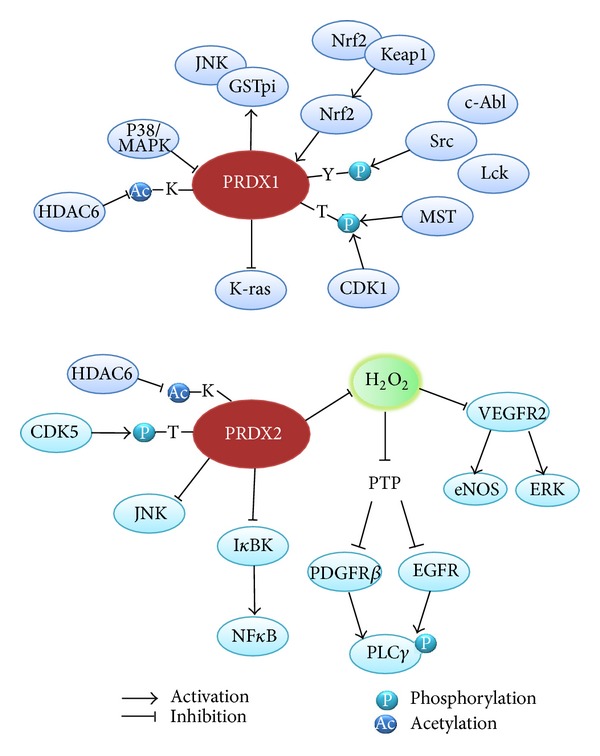
Interaction of typical 2-Cys Prxs with signaling molecules in the phosphorylation and acetylation networks. Prx1 and Prx2 interact directly or indirectly via ROS with the kinases/phosphatases and regulate their activation. In addition, the activities of these two Prxs are also controlled by phosphorylation and acetylation. Note that the Prx1 expression is known to be controlled by a transcription factor Nrf2 (nuclear factor E2-related factor 2) under oxidative stress condition.

Protein phosphorylation is one of the most important posttranslational modifications in the membrane receptor-mediated growth factor and cytokine signaling and as such modulates protein-protein interaction, enzyme activity, and protein stability and structure. Human genome encodes over 500 putative kinase genes and more than 150 protein phosphatases including dual-specificity phosphatases and protein tyrosine phosphatases (PTP). With the exception of the *EYA* subfamily, most protein phosphatases contain a low-pKa cysteine residue at the active site. The sulfhydryl group is thus deprotonated to the thiolate anion at the physiological pH [15, 16], which renders it susceptible to oxidation by H_2_O_2_ *in vitro* and *in vivo *[17, 18]. Since H_2_O_2_ was proposed as a novel intracellular second messenger in the platelet-derived growth factor (PDGF) and epidermal growth factor (EGF) signaling pathways [19, 20], the H_2_O_2_-mediated reversible oxidation of PTPs has become an important regulatory mechanism controlling protein tyrosine phosphorylation [21, 22]. Recently, plausible evidence also indicates that the protein kinases are redox-regulated by a reversible oxidation of the cysteine residues in the regulatory region, rather than on their active sites. For example, I*κ*B kinases *α*/*β* (IKK*α*/*β*) were shown to harbor a reactive cysteine between two serine residues, which are the dual phosphorylation sites critical for activation, in T-loop [23–25]. The ataxia-telangiectasia mutated (ATM) kinase and a Src kinase Lyn were shown to be activated by a H_2_O_2_-mediated cysteine oxidation [26, 27]. Such evidence concertedly indicates that the phosphorylation signaling network involves redox-regulated kinases/phosphatases and therefore it is associated with the dynamics of intracellular H_2_O_2_ level. In a live cell, the intracellular H_2_O_2_ level is determined by balancing the H_2_O_2_ generators (e.g., mitochondria, oxidases, and heavy metals) and antioxidants (e.g., catalase, glutathione peroxidases, and peroxiredoxins). Among cellular peroxidases, 2-Cys Prxs are the most abundant enzymes and versatile in the subcellular distribution. In particular, the evidence indicates that the two cytosolic forms, Prx1 and Prx2, are likely the key enzymes in the phosphorylation signaling pathway (Figure 1). The first indication of the signaling function of Prx was made in 1998 and showed that the overexpression of Prx1 and Prx2 eliminated intracellular H_2_O_2_ increased by growth factors, such as PDGF-B and EGF, and cytokine tumor necrosis factor (TNF)-*α* [1]. Since then, many investigations indicate the important regulatory role of Prx1/2 in phosphorylation signaling. The Prx1 ablation was shown to result in the Akt hyperactivation in H_2_O_2_-treated cells, but not in PDGF-treated cells [28]. Prx1 interacted with phosphatase and tensin homolog (PTEN) in H_2_O_2_-treated cells and thus promoted the Ras- or ErbB2-drived cell transformation. Hence, it was proposed that Prx1 might contribute someway to the tumorigenesis. However, the function of Prx2 as a signal regulator was initially proposed by the differential regulation of TNF-*α*-induced MAP kinase activation [29]. Also, Prx2 negatively regulates the PDGF-induced tyrosine phosphorylation in fibroblast and vascular smooth muscle cells [30]. In this case, the deletion of Prx2, not Prx1, selectively increased the autophosphorylation of PDGFR*β* only at two tyrosine sites (Y579 and Y857), which was not mimicked by addition of exogenous H_2_O_2_. Such selective regulation was achieved by the stimulation-dependent interaction of Prx2 and PDGFR*β* proteins, which allowed the reactivation of a membrane-associated PTP. This is the first report showing the selective action of endogenous H_2_O_2_ distinguished from the exogenous source of H_2_O_2_. Recently, Prx2 was also shown to preserve the VEGFR2-dependent tyrosine phosphorylation in vascular endothelial cells by protecting the receptor from oxidative inactivation by both the endogenous and exogenous H_2_O_2_ [31]. This function appeared to be due to the colocalization of Prx2 and vascular endothelial growth factor receptor-2 (VEGFR2) in endothelial caveolae. Although the source of endogenous H_2_O_2_ was not identified, it is an important finding that Prx2 functions upstream of the receptor tyrosine kinase whose activity is regulated by an oxidation-sensitive cysteine residue.

The cytosolic 2-Cys Prxs are themselves linked to the phosphorylation networks as their activities are regulated by phosphorylation. Chang et al. reported that the 2-Cys Prxs contain the conserved CDK phosphorylation sequence (Thr^90^-Pro-Arg-Lys), and among them the Prx1 and Prx2 were indeed phosphorylated by Cdk1/Cdc2 [32]. Although such threonine phosphorylation caused the loss of peroxidase activity of both 2-Cys Prxs *in vitro*, it was observed only in Prx1 *in vivo *using the mitotic arrested HeLa cells. However, its biological significance remains unsolved. Other studies also showed that the Prx1 threonine phosphorylation is mediated by serine/threonine kinase Mst1/2 [33, 34]. Similarly, this phosphorylation inactivated the peroxidase activity and therefore resulted in an increase in the intracellular H_2_O_2_ level. In contrast, the serine phosphorylation of Prx1 by a T-cell-originated protein kinase (TOPK) increased the peroxidase activity [35]. TOPK binds to and phosphorylates Prx1 at Ser^32^ *in vitro* and in human melanoma cells. It is noteworthy that the activated TOPK colocalized with Prx1 in nucleus, which is the first indication of nuclear Prx1. Later, both Prx1 and Prx2 were found in the nucleus and, particularly, Prx2 protects the cancer cell death against DNA damaging agents [36]. The threonine phosphorylation of Prx2 correlates with an increased loss of dopaminergic neurons by mitochondrial damage [37]. Interestingly, in this case, the phosphorylation was mediated by Cdk5/p35 and increased in nigral neurons from postmortem tissue of Parkinson's disease patients. Related to Parkinson's disease, there was another interesting report that a mutation of leucine rich repeat kinase 2 (LRRK2), where glycine-2019 is mutated to serine, increased the phosphorylation of a mitochondrial Prx3 [38]. The phosphorylation of Prx3 was associated with the increased cell death in neuronal cells by a mitochondrial stress and significantly detected in Parkinson's disease patients with the LRRK2 mutation. Consequently, the phosphorylation-dependent inactivation of mitochondrial Prx3 and cytosolic Prx2 seems to be coordinately involved in the loss of dopaminergic neuronal cells by mitochondrial damage.

Recently, Prx1 was shown to be phosphorylated at Tyr^194^ by protein tyrosine kinases, such as Lck and Abl, *in vitro* and in various mammalian cells treated with growth factors [39]. This evidence is significant in terms of that the inactivation of Prx1 by phosphorylation in caveolae membrane microdomain could alter the local redox status. Although the authors showed the phosphorylated Prx1 in the margin of healing wounds in C57BL/6 mice, the physiological relevance of the selective Prx1 phosphorylation to wound healing process remained uncertain. It is however clear that the phosphorylation-dependent inactivation takes a physiological advantage of the dynamic regulation linked to the intracellular kinase/phosphatase signaling network compared to Prx inactivation by overoxidation, as the reversal by sulfiredoxin of the latter is a slow reaction requiring an ATP energy demand [40, 41].

How does such phosphorylation regulate Prx peroxidase activity? Phosphorylation at Thr^90^ and phosphorylation Tyr^194^ have both been shown to regulate Prx activity by a similar mechanism, potentially involving a perturbation of the active site conformation after the introduction of a negatively charged phosphate moiety at the vicinity of the active site *C*_*P*_ residue [32, 39]. The crystal structure of Prx1 also suggests that the introduction of negative charges may destabilize Prx1 homodimer further causing the reduction of Prx activity toward H_2_O_2_.

Considering that the 2-Cys Prx isoforms are widely distributed in subcellular compartments, such modification-dependent inactivation of the 2-Cys Prxs may be an important mechanism in determining a localized elevation of H_2_O_2_ levels.

## 3. 2-Cys Prxs in Acetylation Signaling Networks

The reversible acetylation of protein lysine residues is an important posttranslational modification that regulates enzyme activity, protein-protein interaction, and protein conformation [42]. The majority of the initial studies focused on the histone acetylation, which directly regulates gene transcription and chromatin remodeling [43]. Since the microtubule-associated HDAC6 and mitochondrial Sirt3 were discovered [44–46], the reversible acetylation has been considered to be a general modification involved in the cellular signaling.

There are several studies implicating the redox regulation of lysine acetylation network. One study showed that H_2_O_2_ inhibits IL-1*β*-induced HDAC2 activity in airway epithelial cells, which is associated with the tyrosine nitration of HDAC2 [47]. Another study showed that H_2_O_2_ and hypertrophic stimuli induce a cysteine oxidation on HDAC4 in myocytes [48]. Upon oxidation, HDAC4 forms an intramolecular disulfide linkage and then the oxidized HDAC4 is exported to the cytoplasm. When the disulfide was reduced by Trx1, the reduced HDAC4 reenters into the nucleus. Consequently, the nucleocytoplasmic shuttling of HDAC4 is determined by its cysteine oxidation status. A member of class II HDACs, Sirt1, was shown to be sensitive to oxidation, especially S-glutathionylation on the Cys^67^ residue by S-nitrosoglutathione (GSNO) [49]. Interestingly, the GSNO inhibited the resveratrol-stimulated, not the basal, Sirt1 activity, which suggests that the redox-sensitive Cys residue could be exposed to the modification upon activation. The Sirt3 knockout mice showed oxidative stress phenotype in skeletal muscle and its knockdown in cultured myoblasts increased the ROS level [50].

Many studies show that the 2-Cys Prx activity is regulated by acetylation (Figure 1). A recent high-resolution mass spectrometric analysis combined with the stable isotope labeling by amino acids in cell culture (SILAC) revealed the lysine acetylation of Prx enzymes in various cell types [51]. A previous study showed that the Prx1 and Prx2 were among the substrates of cytoplasmic HDAC6 and their acetylation increased peroxidase activity and resistance to overoxidation [52]. It was shown that a lysine residue in the C-terminus of Prx1 and Prx2 enzymes (Lys197 in Prx1 and Lys196 in Prx2) is a site of acetylation. Thus, although the molecular mechanism underlying the acetylation-dependent activity increase is currently unknown, it is possible that the C-terminal acetylation may influence the resolving step accompanied with a conformation change of the *C*_*R*_ residue [9]. In the case of Prx2, the lysine-independent acetylation at its demethionylated N-terminus conferred a resistance to overoxidation in HeLa cells treated with high concentrations of H_2_O_2_ [53]. It is noteworthy that the acetylation of 2-Cys Prxs increases the enzyme activity and protects against overoxidation in contrast to enzyme inactivation by phosphorylation.

Although there is no evidence showing a direct regulatory role of 2-Cys Prxs in the lysine acetylation network, it will be interesting to investigate the mechanism of how the acetylation and deacetylation network is associated with 2-Cys Prxs in various subcellular compartments.

## 4. 2-Cys Prxs in Cell Death Signaling Networks

The role of ROS in cell death has been a long-standing issue because mitochondria are the key players in both apoptotic and necrotic cell death pathways. Indeed, mitochondria are the site where the electron transport takes place and leakage of the high energy electrons from the electron carrier complexes can combine with molecular oxygen to produce ROS [54]. Higher organisms with an aerobic respiratory system have evolved apoptotic cell death programs utilizing mitochondrial proteins, which include cytochrome c [55, 56]. In principle, the mitochondrial release of cytochrome c results in a disruption of the electron transport in the respiratory chain and causes an increase of mitochondrial ROS via the leakage of high energy free electrons. The resulting ROS burst may oxidatively damage the cellular macromolecules, such as proteins, membrane lipids, and DNA. However, the evidence indicates that the mitochondrial ROS is not a causative factor in apoptotic cell death, but rather it is the consequence of the disruption of mitochondrial transmembrane potential (Δ*ψ*_*m*_) [57]. The involvement of ROS in the apoptotic death pathway could be challenged by the fact that the active site of caspase is a reactive cysteine residue, which can be inactivated by oxidation [58–60]. Contrary to apoptosis, there may be a function of ROS in necroptosis. Necroptosis, also called programmed necrosis, is a type of necrotic cell death involving the activation of death receptor but occurring independently of caspase activation [61]. It has been shown that activation of death receptors, such as the TNF-*α* receptor (TNFR)-1 and Fas (CD95), induces necroptosis in some cell types [62, 63]. For example, mouse fibrosarcoma cells L929 underwent caspase-independent necrosis when stimulated with TNF-*α* [64]. Human Jurkat T lymphoma cells deficient in Fas-associated death domain (FADD) adaptor protein died via necrosis when death receptors, such as TNFR and Fas, were activated in an RIP1-dependent manner [65]. Therefore, the necroptosis was found to require the RIP1 kinase activity [66]. Further evidence indicates that ROS accumulates in a RIP1 and FADD-dependent manner and is required for the necroptosis [67]. The activation of NADPH oxidase-1 via RIP1 is involved in the TNF-*α*-induced necrosis in L929 cells [68]. In addition, RIP3, which was shown to be the most essential factor for necroptosis [69, 70], was involved in the production of mitochondrial ROS via energy metabolism [71].

**Figure 2:**
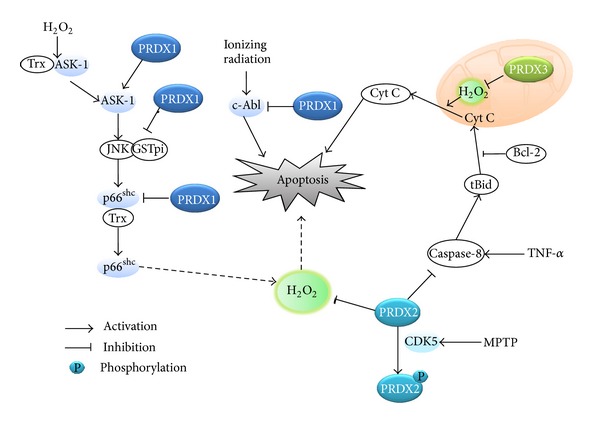
Role of typical 2-Cys Prxs in the apoptotic death pathways. The schematic drawing illustrates that each Prx interacts with proapoptotic molecules and regulates various apoptotic pathways. MPTP: 1-methyl-4-phenyl-1,2,3,6-tetrahydropyridine.

The cell death studies by modulation of cellular antioxidant enzymes reveal a clear role of intracellular ROS in apoptosis. Particularly, the 2-Cys Prxs play a regulatory role in apoptotic, not necrotic, cell death (Figure 2). Prx1 was shown to protect lung cancer cells from radiation-induced apoptotic cell death by reducing JNK activation [72]. Interestingly, Prx1 prevented the JNK activation by retaining the JNK associated with glutathione S-transferase (GST)-pi, but not through the peroxidase activity. It was also shown that the expression of Prx1 in dopaminergic neuronal cells inhibited 6-hydroxydopamine-induced apoptotic death by reducing the p38/caspase-3 activation [73]. The level of Prx1 was obviously upregulated in human lung cancer patients and the Prx1 knockdown in hepatocarcinoma cells accelerated the TNF-related apoptosis-inducing ligand (TRAIL)-induced cell death via caspase-8/-3 activation [74]. Prx1 also mediated the disulfide-linked activation of the apoptosis signaling kinase ASK1 by forming a mixed disulfide intermediate with ASK1 in the peroxide-treated cells [75]. It has been shown that Prx2 and Prx3 reduce apoptotic cell death via mitochondrial-dependent intrinsic pathway [76, 77]. Interestingly, the redox cycle of the Prx3 activity shifted to the disulfide-containing oxidized state during Fas-mediated apoptosis of Jurkat and U937 monocytic cells [78]. Collectively, the evidence related to the 2-Cys Prxs strongly indicates that ROS is connected to the apoptotic cell death. Further exploration is needed to determine the molecular mechanism underlying antiapoptotic role of 2-Cys Prxs.

## 5. Signaling Role of 2-Cys Prxs Beyond Peroxidase Enzyme

Despite the 2-Cys Prx being a sophisticated peroxidase enzyme with a high affinity to H_2_O_2_ [79], recent studies also suggest that 2-Cys Prx can function as redox protein that regulates the activity of various client proteins by direct protein-protein interaction or interprotein disulfide linkage. In 1997, it was reported that Prx1 interacts with the SH3 domain of c-Abl and inhibits its tyrosine kinase activity [80]. It was the first report showing that the 2-Cys Prx is one of the redox proteins capable of regulating a key signaling kinase. Subsequently, Prx1 has been found to interact with the Myc Box II (MBII) domain of c-Myc by a yeast two-hybrid screen [81]. By this interaction, Prx1 contributed to an antioxidative stress function and it did also inhibit the c-Myc-dependent target gene expression and tumorigenesis. Park and her colleagues showed that Prx1 interacts with androgen receptor in various prostate cancer cell lines and GST-pi in lung cancer cell lines [72, 82]. The Prx1 interaction with androgen receptor promotes the receptor's transactivation activity. Later, it turned out that Prx1 increases the receptor affinity to dihydrotestosterone [83]. The findings seem to be important in relation to the high Prx1 expression in the prostate cancer patients [84]. Another interesting result was that Prx1 interacts and forms a mixed disulfide linkage with the GDE2 activation in spinal motor neurons [85]. In motor neuron progenitors, Prx1 promotes the GDE2 activity to drive a neuronal differentiation by reducing an intramolecular disulfide linkage in the cytoplasmic tail of the transmembrane protein. This evidence indicates that the Prx1 can function as a protein disulfide reductase (PDI). Other example of PDI activity among 2-Cys Prxs is Prx4 in endoplasmic reticulum. It was shown that the oxidized Prx4 transfers the disulfides to PDI [5, 6]. The Prx4 reoxidation is achieved by metabolizing H_2_O_2_ produced by Ero1, which is known as the main ER enzyme responsible for reoxidation of protein disulfide reductase [86]. This evidence defines a new role of Prx4 in oxidative protein folding along with Ero1.

In contrast to the case of Prx1, the closest isoform Prx2 has barely been shown to directly interact with any protein. Actually, few reports are stating that Prx2 colocalizes and interacts with phospholipase D1 in phorbol ester-stimulated cells [87] as well as interacts with the PDI family member, ERp46, when under its overoxidized form [88]. The *in vitro* activity assays showed that Prx2 is less active as a peroxidase enzyme than Prx1 [1, 39]. Given the *in vitro* evidence that the 2-Cys Prxs is inactivated by overoxidation during the reaction cycle proportional to the enzyme activity [8], it is conceivable that Prx1 is the peroxidase enzyme acting as the first line of antioxidant defense under H_2_O_2_ stress. Nonetheless, it turned out that Prx2 is more susceptible for overoxidation in the animal cells under H_2_O_2_ stress than Prx1 [39]. The same study shows that Prx1 rather prefers to be tyrosine phosphorylated under H_2_O_2_ stress *in vivo*. This discrepancy between *in vitro *and* in vivo* properties of Prx1 and Prx2 could be explained as a paradox: unlike the potential function assumed from the *in vitro* characterization, the Prx2 can be the real peroxidase enzyme in the cells while the Prx1 primarily functions as a redox regulator of diverse client proteins by interaction. This idea is supported to some extent because it was observed that Prx1 was more abundant in protein amount than was Prx2 in certain cell types like fibroblasts and HeLa cells.

**Figure 3:**
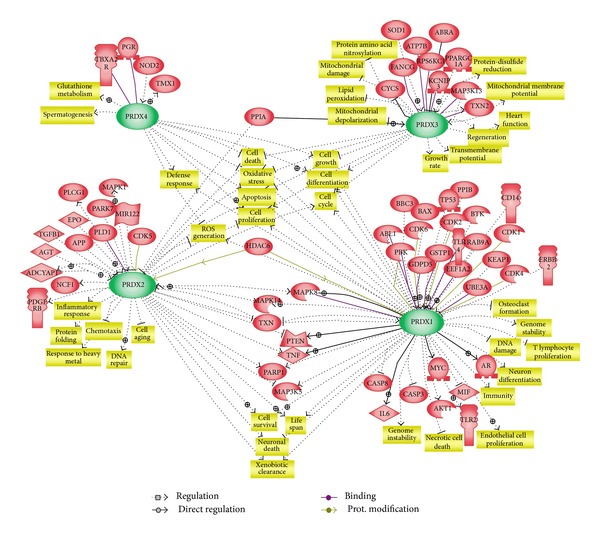
Summary of regulatory networks centered by typical 2-Cys Prxs. Functional correlation and interaction among the signaling proteins and typical 2-Cys Prxs are reconstituted into a network model using the *Pathway Studio* software (Ariadne Genomics Inc., USA). All molecules are shown as gene symbol. Direct and indirect regulations were indicated in gray lines and dotted gray lines, respectively. Green arrows indicate regulation by protein modifications. Purple lines indicate direct protein-protein binding.

In an effort to obtain a global picture of regulations by 2-Cys Prxs, we finally produced a network model among typical 2-Cys Prxs using the *Pathway Studio* software. We obtained the network relations for all four Prxs in the knowledge base of Pathway Studio, which were built by text-mining of literature texts. False positives or indirect relations were removed by inspecting the relevant sentences manually.  Figure 3 shows the resulting network model where relations specific to each Prx were located near the corresponding Prx and entities involved in more than one Prxs were positioned in the intervening space. This network includes the direct interaction of 2-Cys Prxs and their client proteins as mentioned above. As expected, it is evident that all four Prxs are closely related to apoptosis and cell death, ROS generation and oxidative stress, cell proliferation, growth, and differentiation. This diagram also illustrates biological processes and functions specific to each Prx or common between two Prxs. For example, Prx3 is specifically related to mitochondrial damage and lipid peroxidation. It can be readily seen that cytosolic enzymes Prx1 and Prx2 are related to cell survival via PTEN, TNF, MAP kinases, and PARP1. Overall, the network model emphasizes the importance of typical 2-Cys Prxs as hub molecules connecting cellular signaling pathways and biological processes.

## 6. Conclusion Remarks

Four members of typical 2-Cys Prx subfamily are present in various cellular compartments, including cytosol, plasma membrane (especially caveolae), nuclei, mitochondria, and endoplasmic reticulum. The majority of the abundant 2-Cys Prx enzymes primarily function as general antioxidant systems that maintain the intracellular ROS level within a safety zone in both normal and stressed cells. However, some part of the enzymes functions as the signal regulator at specific locations by modulating the local ROS change or by regulating the activity of the interacting/neighboring proteins in a redox-dependent manner. Since H_2_O_2_ is an important second messenger in a signaling network, the discovery of the 2-Cys Prx function related to signal transduction should provide clues necessary to understand redox signaling architecture and further solve medical problems in ROS-mediated chronic diseases.
